# Perioperative Determinants of Postoperative Morbidity After Liver Resection: From Morphofunctional Vulnerability to Inflammatory Response

**DOI:** 10.3390/jcm15103581

**Published:** 2026-05-07

**Authors:** Leticia Pérez-Santiago, Isabel Mora-Oliver, Marina Garcés-Albir, Elena Muñoz-Forner, Luis Sabater-Ortí, Dimitri Dorcaratto

**Affiliations:** 1 Colorectal Surgery Unit, Department of General and Digestive Surgery, Hospital Clínico Universitario, INCLIVA Biomedical Research Institute, 46010 Valencia, Spain; 2 The Hospital Clínico Universitario de Valencia, Avenida Blasco Ibáñez No. 17, 46010 Valencia, Spain; 3 Liver-Biliary and Pancreatic Surgery Unit, Department of General and Digestive Surgery, Hospital Clínico Universitario, INCLIVA Biomedical Research Institute, 46010 Valencia, Spain; 4 Department of Anatomy, University of Valencia, 46010 Valencia, Spain; 5 Department of Surgery, University of Valencia, 46010 Valencia, Spain

**Keywords:** liver resection, perioperative risk, skeletal muscle index, malnutrition, inflammatory response, postoperative complications, prehabilitation

## Abstract

**Background:** Postoperative morbidity after liver resection remains considerable despite advances in surgical and perioperative care. Risk stratification has traditionally focused on surgical factors, although patient-related vulnerability and the postoperative inflammatory response may also play a critical role. This study aimed to evaluate the relative contribution of perioperative factors, including morphofunctional vulnerability (skeletal muscle index [SMI] and GLIM-defined malnutrition), surgical variables, and inflammatory biomarkers, in predicting postoperative outcomes after liver resection. **Methods:** We conducted a retrospective single-center study including patients who underwent liver resection between 2017 and 2021. Preoperative morphofunctional assessment included skeletal muscle index (SMI) measured at the L3 level on computed tomography and nutritional status defined according to GLIM criteria. Perioperative variables included surgical factors and postoperative inflammatory and immune biomarkers. Outcomes were overall complications within 90 days, major complications (Clavien–Dindo ≥ III), and severe morbidity defined by a Comprehensive Complication Index (CCI) ≥ 26.2. Multivariable logistic regression analyses were performed to identify independent predictors. **Results:** A total of 253 patients were analysed. Overall complications occurred in 35.6% of patients, with major complications in 14.6% and severe morbidity in 17.4%. Low SMI was present in 56.1% of patients. In multivariable analysis, low SMI independently predicted postoperative complications (OR 2.23, 95% CI 1.14–4.38; *p* = 0.02), along with the open surgical approach, operative time ≥ 193 min, and elevated C-reactive protein on postoperative day 3. For major complications (Clavien–Dindo ≥ III), low SMI remained a strong independent predictor (OR 4.28, 95% CI 2.76–10.40; *p* = 0.001), together with surgical factors and lymphopenia on postoperative day 3. In contrast, GLIM-defined malnutrition was independently associated with severe morbidity (CCI ≥ 26.2) (OR 2.95, 95% CI 1.44–6.06; *p* = 0.003). **Conclusions:** Postoperative morbidity after liver resection is determined by a combination of perioperative factors, including patient-related morphofunctional vulnerability, surgical complexity, and postoperative inflammatory response. Skeletal muscle depletion is primarily associated with the occurrence of complications, whereas GLIM-defined malnutrition appears to influence their severity. These findings support a comprehensive perioperative approach to risk stratification and highlight the potential role of targeted prehabilitation strategies aimed at improving surgical resilience and postoperative outcomes.

## 1. Introduction

Liver resection remains the cornerstone curative treatment for a wide range of primary and secondary hepatic malignancies. Over the past decades, advances in surgical techniques, perioperative care, and enhanced recovery after surgery (ERAS) protocols have significantly reduced perioperative mortality. However, postoperative morbidity after hepatectomy remains considerable, with complication rates reported between 20% and 40% even in high-volume centres. These complications may adversely affect short-term recovery, prolong hospital stay, increase healthcare costs, and delay or even prevent the administration of adjuvant oncological treatments [1,2].

Traditional preoperative risk stratification in liver surgery has focused mainly on surgical complexity, the extent of resection, and baseline comorbidities. Although these factors undoubtedly influence postoperative outcomes, they may not fully capture the patient’s physiological capacity to tolerate major surgical stress. Increasing attention has therefore been directed toward the concept of physiological reserve, which reflects the organism’s ability to respond to the metabolic and inflammatory demands associated with major surgery [3].

Among the different determinants of physiological reserve, body composition has emerged as a key factor in oncologic surgery. Skeletal muscle plays a central role in metabolic regulation, protein turnover, and immune function [4]. Reduced skeletal muscle mass, commonly referred to as sarcopenia, has been associated with impaired metabolic reserve, increased inflammatory response, and worse postoperative outcomes across multiple surgical specialties. In liver surgery specifically, sarcopenia has been linked to an increased risk of postoperative complications and poorer clinical outcomes [5].

Computed tomography (CT), routinely performed for oncologic staging and surgical planning, allows an objective assessment of skeletal muscle mass through measurement of the muscle area at the level of the third lumbar vertebra. The skeletal muscle index (SMI), obtained by normalizing muscle area to patient height, has been widely used as a reproducible indicator of muscle depletion [6]. This approach enables the integration of body composition analysis into routine preoperative evaluation without additional tests or patient burden.

In addition to body composition, nutritional status has been increasingly recognized as a major determinant of surgical outcomes [7]. Disease-related malnutrition (DRM) is common among patients undergoing oncologic surgery and is associated with impaired immune function, delayed wound healing, and increased postoperative morbidity [8]. The Global Leadership Initiative on Malnutrition (GLIM) has proposed a standardized diagnostic framework that combines phenotypic criteria such as weight loss or low body mass index with etiological factors including reduced food intake and inflammatory burden, allowing a more comprehensive evaluation of nutritional status in surgical populations [9].

Assessment of postoperative outcomes also requires standardized and reproducible measures of complication severity. The Clavien–Dindo classification is widely used to grade postoperative complications according to the level of intervention required [10]. In addition, the Comprehensive Complication Index (CCI) provides a continuous score that integrates all postoperative complications into a single numerical value, offering a more accurate representation of the global burden of postoperative morbidity [11].

Importantly, both skeletal muscle depletion and malnutrition represent potentially modifiable vulnerability factors. These concepts have gained particular relevance in the context of perioperative optimization and prehabilitation strategies, which aim to improve functional capacity and metabolic reserve before major surgery. Early identification of high-risk patients may therefore allow the implementation of targeted interventions, including exercise training, nutritional optimization, and multimodal prehabilitation programs aimed at improving surgical resilience. The identification of modifiable risk factors such as sarcopenia and malnutrition has increased interest in perioperative optimization strategies, including multimodal prehabilitation programs [12].

Within this framework, identifying patients with reduced physiological reserve before surgery has become increasingly important to guide perioperative optimization strategies. A growing body of evidence has demonstrated that alterations in nutritional status and body composition -particularly sarcopenia- are associated with an increased risk of postoperative outcomes, prolonged hospital stay, and worse oncological outcomes after liver resection [13,14]. However, despite this accumulating evidence, the integration of these parameters into routine preoperative risk stratification remains inconsistent, and their relative contribution when different measures of postoperative morbidity are considered has not been fully clarified.

Therefore, the aim of the present study was to investigate the role of perioperative factors in predicting postoperative outcomes after liver resection, with a particular focus on preoperative morphofunctional vulnerability—specifically skeletal muscle depletion assessed by computed tomography-derived skeletal muscle index (SMI) and GLIM-defined malnutrition—alongside surgical variables and postoperative inflammatory response. In addition, we aimed to determine whether these markers of physiological reserve were differentially associated with distinct measures of postoperative complications, including overall complications, major complications according to the Clavien–Dindo classification, and severe morbidity quantified by the Comprehensive Complication Index.

## 2. Materials and Methods

We conducted a retrospective, observational, single-centre study including consecutive adult patients who underwent liver resection at the Hospital Clínico Universitario de Valencia between January 2017 and September 2021. The study was designed and reported according to the STROBE (Strengthening the Reporting of Observational Studies in Epidemiology) statement.

Adult patients undergoing liver resection for benign or malignant hepatic lesions during the study period were eligible for inclusion. Patients were excluded if intraoperative findings revealed unresectable disease, if the target lesion could not be identified during surgery, or if preoperative CT imaging suitable for body composition analysis was not available. Only patients with complete clinical and imaging data allowing evaluation of body composition and postoperative outcomes were included in the final analysis.

Clinical, demographic, and perioperative variables were obtained from electronic medical records and institutional databases. Baseline variables included age, sex, body mass index (BMI), comorbidities, Charlson comorbidity index, and American Society of Anesthesiologists (ASA) physical status classification.

Surgical variables included type and extent of liver resection, operative approach, operative time, and relevant intraoperative parameters.

Postoperative outcomes included overall complications within 90 days after surgery, major complications defined as Clavien–Dindo grade ≥ III, and severe morbidity quantified using the Comprehensive Complication Index (CCI), with a threshold of ≥26.2.

### 2.1. CT-Based Body Composition Analysis

Body composition was assessed using preoperative CT scans performed as part of routine preoperative evaluation.

Skeletal muscle mass was measured at the level of the third lumbar vertebra (L3) using manual and semi-automatic segmentation with ImageJ software M14.1 (National Institutes of Health, Bethesda, MD, USA). Muscle tissue was identified using attenuation thresholds ranging from −29 to +150 Hounsfield units, following previously described methodology for CT-based body composition analysis [15].

The cross-sectional skeletal muscle area (cm^2^) was calculated and normalized for patient height to obtain the SMI (cm^2^/m^2^).

All CT images were extracted in anonymized DICOM format, and image analysis was performed by a trained investigator to ensure consistency and minimize intra-observer variability. CT scans were considered unsuitable if they did not include the L3 level or had inadequate image quality for segmentation.

Low skeletal muscle mass was defined according to the cut-off values proposed by Dolan et al. [16] which stratify the skeletal muscle index according to sex and body mass index. The following thresholds were applied: <45 cm^2^/m^2^ for men with BMI < 25 kg/m^2^, <53 cm^2^/m^2^ for men with BMI ≥ 25 kg/m^2^, <39 cm^2^/m^2^ for women with BMI < 25 kg/m^2^, and <41 cm^2^/m^2^ for women with BMI ≥ 25 kg/m^2^.

Additionally, BMI-adjusted thresholds based on BMI ≥ 30 kg/m^2^ were also considered according to the original publication (<45.6 cm^2^/m^2^ and <56.8 cm^2^/m^2^ for men; <39.1 cm^2^/m^2^ and <44.6 cm^2^/m^2^ for women, for BMI < 30 kg/m^2^ and ≥30 kg/m^2^, respectively).

To account for potential population-specific differences, cohort-specific cut-off values for skeletal muscle index were derived using receiver operating characteristic (ROC) curve analysis, selecting the optimal threshold according to the Youden index for the prediction of postoperative complications.

For multivariable modelling, only ROC-derived cut-offs were used, while literature-based thresholds were considered for descriptive and exploratory analyses.

Sarcopenic obesity was defined as the coexistence of obesity and low skeletal muscle mass [17]. Obesity was defined as a body mass index (BMI) ≥ 30 kg/m^2^, and low skeletal muscle mass was defined according to the same BMI- and sex-adjusted SMI cut-off values proposed by Dolan et al. [16].

### 2.2. Nutritional Assessment

Nutritional status was assessed according to the GLIM criteria [9]. The diagnosis of malnutrition required the presence of at least one phenotypic criterion and one etiologic criterion.

In this study, phenotypic criteria included low SMI assessed by computed tomography and low BMI. Weight loss was not included as a phenotypic criterion due to the absence of systematically recorded data in the retrospective dataset. As etiologic criteria, the presence of disease-related inflammation associated with the oncologic condition or chronic inflammatory comorbidities documented in the medical records was considered.

Low skeletal muscle mass used as a phenotypic criterion within the GLIM framework was defined according to the same cut-off values proposed by Dolan et al. [16].

Cut-off values proposed by Dolan et al. [16] were selected because they were derived from a European population and stratified by BMI, allowing better identification of sarcopenia in patients with overweight or obesity.

### 2.3. Statistical Analysis

Continuous variables were expressed as mean ± standard deviation or median with interquartile range (IQR) depending on data distribution, while categorical variables were presented as frequencies and percentages.

Comparisons between groups were performed using Student’s *t* test or the Mann–Whitney U test for continuous variables and the χ^2^ test or Fisher’s exact test for categorical variables, as appropriate.

Receiver operating characteristic (ROC) curve analysis was used to evaluate the discriminative ability of continuous variables for postoperative complications. Optimal cut-off values were determined using the Youden index.

Variables with *p* < 0.10 in univariable analysis were included in multivariable logistic regression models to identify independent predictors of postoperative complications.

Collinearity between low SMI and GLIM-defined malnutrition was low, with VIF values of 3.5 and 4.1, respectively, indicating no relevant multicollinearity.

A complete case analysis was performed, and only patients with complete clinical and imaging data were included in the final analysis.

Statistical significance was defined as *p* < 0.05. All statistical analyses were performed using IBM SPSS Statistics (version 23, IBM Corp., Armonk, NY, USA).

### 2.4. Ethical Considerations

This study was conducted in accordance with the principles of the Declaration of Helsinki.

The study protocol was approved by the Ethics Committee of the Hospital Clínico Universitario de Valencia. Due to the retrospective design of the study and the use of anonymized clinical data, the requirement for written informed consent was waived.

## 3. Results

**Figure 1:**
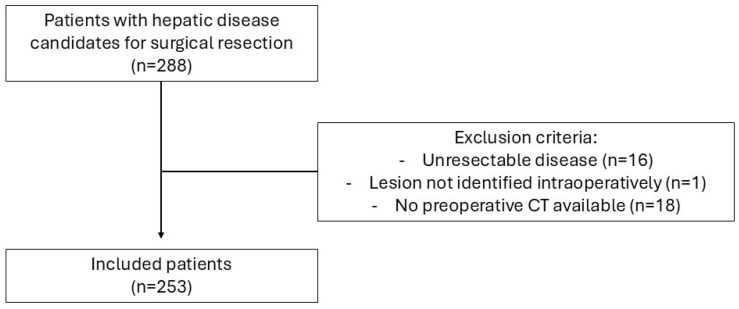
Flow diagram of patient selection.

During the study period, 288 patients with benign or malignant hepatic lesions were assessed as potential candidates for liver resection. Of these, 16 patients were excluded due to intraoperative confirmation of unresectable disease, one patient was excluded because the target lesion was not identified during surgery, and 18 patients were excluded due to the absence of suitable preoperative computed tomography (CT) imaging. A total of 253 patients were ultimately included in the final analysis (Figure 1).

Baseline clinical, nutritional, and morphofunctional characteristics are summarized in Table 1. The median age was 65 years (IQR 58–73), and 61.7% of patients were male. The median BMI was 26.3 kg/m^2^ (IQR 23.6–29.7), and most patients were classified as ASA ≥ III (59.3%), with a median Charlson comorbidity index of 7 (IQR 6–8).

Low skeletal muscle mass was highly prevalent, affecting 56.1% of patients according to BMI-adjusted cut-offs. Notably, median SMI values were slightly higher in patients who developed complications, although this difference did not reach statistical significance (*p* = 0.08). When applying literature-based cut-offs (Dolan et al. [16]), low SMI showed a non-significant trend toward higher rates of postoperative complications (BMI ≥ 25: 60.1% vs. 48.9%, *p* = 0.08; BMI ≥ 30: 54% vs. 42.2%, *p* = 0.07).

ROC curve analysis was performed to determine cohort-specific SMI cut-offs, which showed modest but clinically relevant discriminatory capacity. The optimal thresholds were ≤45.7 cm^2^/m^2^ for men and ≤35.96 cm^2^/m^2^ for women and were used for subsequent multivariable analyses.

The prevalence of malnutrition varied substantially depending on the GLIM definition applied. When defined using BMI alone, malnutrition was identified in 11.9% of patients, whereas the inclusion of muscle mass as a phenotypic criterion increased the prevalence to over 50%. Interestingly, GLIM-defined malnutrition appeared to be more prevalent among patients without postoperative complications, although this difference did not reach statistical significance, suggesting a potential paradoxical association in univariable analysis.

Tumour and surgical characteristics are summarized in Table 2. The majority of resections were performed for malignant disease (93.3%), predominantly colorectal liver metastases (66.8%). The median number of lesions was 1 (IQR 1–12), with a median tumour size of 23 mm (IQR 4–180).

An open approach was used in 67.6% of cases, and 21.7% of patients underwent major hepatectomy. The median operative time was 210 min (IQR 45–510), and extrahepatic procedures were performed in 24.5% of patients.

In univariable analysis, posterior segment involvement (*p* = 0.04), preoperative embolization (*p* = 0.004), extrahepatic procedures (*p* = 0.01), operative time (*p* = 0.001), and open surgical approach (*p* < 0.001) were significantly associated with postoperative complications.

Postoperative inflammatory and immune biomarkers are summarized in Table 3. C-reactive protein levels on postoperative day 3 were significantly higher in patients who developed complications (*p* = 0.02), while lymphocyte count on postoperative day 3 was significantly lower in this group (*p* = 0.001).

In addition, the neutrophil-to-lymphocyte ratio was significantly elevated in patients with complications on postoperative days 1 and 3 (*p* = 0.03 and *p* = 0.02, respectively). No significant differences were observed for leukocyte count, platelet-to-lymphocyte ratio, or lymphocyte-to-monocyte ratio.

Postoperative outcomes are presented in Table 4. Overall complications occurred in 35.6% of patients within 90 days, while major complications (Clavien–Dindo ≥ III) were observed in 14.6%. Severe morbidity, defined as a Comprehensive Complication Index (CCI) ≥ 26.2, occurred in 17.4% of patients.

Reintervention was required in 11.1% of cases. The median length of hospital stay was 5 days (IQR 1–90), and both 30- and 90-day mortality rates were 1.6%.

Multivariable analysis is presented in Table 5. Both patient-related and perioperative factors were independently associated with postoperative complications. Low skeletal muscle index emerged as an independent predictor (OR 2.23, 95% CI 1.14–4.38; *p* = 0.02). Surgical factors, including open approach (OR 3.14, 95% CI 1.47–6.70; *p* = 0.003) and operative time ≥ 193 min (OR 3.07, 95% CI 1.58–5.98; *p* = 0.001), were also significant contributors. In addition, elevated C-reactive protein levels on postoperative day 3 (≥124 mg/L) were associated with an increased risk of complications (OR 2.07, 95% CI 1.10–3.91; *p* = 0.02).

The multivariable model for severe postoperative morbidity showed acceptable discriminatory capacity (AUC 0.74, 95% CI 0.67–0.81; *p* < 0.001) and good calibration (Hosmer–Lemeshow *p* = 0.61).

To further explore the determinants of complication severity, separate multivariable models were constructed for major complications (Clavien–Dindo ≥ III) and severe morbidity (CCI ≥ 26.2).

In the multivariable model for major complications (Clavien–Dindo ≥ III) (Table 6), diabetes mellitus (OR 2.80, 95% CI 1.11–7.04; *p* = 0.02), low skeletal muscle index (OR 4.28, 95% CI 2.76–10.40; *p* = 0.001), tumour size ≥ 20.5 mm (OR 3.20, 95% CI 1.24–8.23; *p* = 0.01), open surgical approach (OR 4.89, 95% CI 1.51–15.85; *p* = 0.008), posterior segment involvement (OR 2.78, 95% CI 1.18–6.52; *p* = 0.01), operative time ≥ 185 min (OR 2.81, 95% CI 1.05–7.47; *p* = 0.03), and lymphocyte count < 0.7 ×10^9^/L on postoperative day 3 (OR 4.93, 95% CI 1.90–12.78; *p* = 0.001) were identified as independent predictors.

The multivariable model for major complications (Clavien–Dindo ≥III) showed good discriminatory capacity (AUC 0.83, 95% CI 0.78–0.89; *p* < 0.001) and good calibration (Hosmer–Lemeshow *p* = 0.70).

In the multivariable analysis for severe postoperative morbidity defined as CCI ≥ 26.2 (Table 7), GLIM-defined malnutrition emerged as an independent predictor (OR 2.95, 95% CI 1.44–6.06; *p* = 0.003). In addition, surgical factors including posterior segment involvement (OR 2.33, 95% CI 1.13–4.80; *p* = 0.02) and major hepatectomy (OR 2.46, 95% CI 1.13–5.34; *p* = 0.02) were significantly associated with increased risk of severe complications.

The multivariable model for severe postoperative morbidity (CCI ≥ 26.2) showed acceptable discriminatory capacity (AUC 0.72, 95% CI 0.64–0.80; *p* < 0.001) and good calibration (Hosmer–Lemeshow *p* = 0.92).

ROC curves for the multivariable models are provided in the Supplementary Materials (Figures S1–S3).

## 4. Discussion

Postoperative morbidity after liver resection remains a significant clinical challenge despite advances in surgical techniques and perioperative care. The findings of our study suggest that preoperative morphofunctional vulnerability, particularly reduced skeletal muscle mass assessed by SMI, is independently associated with postoperative complications. Furthermore, our findings show that different components of vulnerability are differentially associated with distinct measures of postoperative morbidity. While skeletal muscle depletion was consistently associated with overall and major complications, GLIM-defined malnutrition was more strongly associated with severe morbidity as assessed by the CCI.

These results support the concept that traditional risk factors alone are insufficient to fully capture surgical risk. Although operative approach and surgical complexity remain key determinants of postoperative outcomes, they do not adequately reflect the patient’s physiological reserve. Increasing evidence suggests that body composition plays a crucial role in determining the ability to respond to surgical stress, particularly in oncologic populations [18,19]. In this context, skeletal muscle has emerged as a central component of metabolic and immunological resilience [20].

Our findings are consistent with previous studies reporting an association between sarcopenia and adverse outcomes after liver resection. Several authors have demonstrated that reduced skeletal muscle mass is associated with increased postoperative complications, prolonged hospital stay, and impaired recovery [20,21]. CT-based assessment of skeletal muscle at the L3 level is currently considered the reference method for evaluating muscle depletion, offering a reproducible and objective measure that can be easily integrated into routine preoperative imaging [22].

Importantly, our study extends previous literature by incorporating GLIM-defined malnutrition into the analysis. Unlike isolated measures of muscle mass, GLIM criteria provide a multidimensional assessment of nutritional status by combining phenotypic and etiologic components [9]. In our cohort, GLIM-defined malnutrition was not independently associated with overall complications but emerged as a significant predictor of severe morbidity (CCI ≥ 26.2). This finding suggests that global nutritional impairment may be more closely related to the cumulative burden and severity of complications rather than their mere occurrence.

From a pathophysiological perspective, these results are biologically plausible. Skeletal muscle plays a key role in protein metabolism, glucose homeostasis, and immune function. Muscle depletion has been associated with impaired anabolic capacity, increased catabolic signaling, and dysregulated inflammatory responses [23]. In line with this, postoperative inflammatory and immune biomarkers, including C-reactive protein and lymphocyte count, were independently associated with postoperative complications. Clinically relevant threshold values were identified, suggesting that these markers may help stratify the risk of postoperative complications. However, these biomarkers should be interpreted as early indicators of evolving postoperative complications and of the host response to surgical stress, rather than true independent predictors. Their association with outcomes likely reflects early manifestations of complications rather than a direct causal relationship.

Interestingly, our results showed an apparent discrepancy between univariable and multivariable analyses regarding morphofunctional parameters. In univariable comparisons, skeletal muscle index (SMI) and GLIM-defined malnutrition were not consistently associated with the occurrence of complications, and in some cases showed paradoxical trends. However, after adjustment for confounding factors, low SMI emerged as an independent predictor of postoperative complications, while GLIM-defined malnutrition was associated with complication severity.

This apparent inconsistency may be explained by several factors. First, univariable analyses do not account for the complex interplay between surgical factors, inflammatory response, and patient-related characteristics, which may confound the crude association between body composition and outcomes. In our cohort, variables such as surgical approach, operative time, and tumour-related factors likely influenced these relationships.

Second, the use of predefined cut-off values for skeletal muscle index may not be fully applicable across different populations. Interestingly, literature-based SMI cut-offs did not reach statistical significance in our cohort, despite showing a consistent trend toward higher complication rates. In contrast, ROC-derived thresholds demonstrated improved discriminatory capacity. This finding highlights the potential limitations of applying universal cut-offs across heterogeneous populations and supports the use of population-specific thresholds to better identify clinically relevant muscle depletion. Importantly, GLIM criteria were not modified per se; rather, the definition of low skeletal muscle mass within the GLIM framework was adapted using these cohort-specific thresholds.

Third, GLIM-defined malnutrition captures a broader and more complex construct of physiological vulnerability, integrating both phenotypic and etiological components. In our study, the absence of weight loss data may have influenced its performance in predicting overall complications. However, its strong association with severe morbidity supports its role as a marker of reduced resilience and impaired recovery capacity rather than the mere occurrence of complications.

Taken together, these findings suggest that skeletal muscle depletion and GLIM-defined malnutrition reflect different, yet complementary, dimensions of morphofunctional vulnerability. While SMI appears to be more closely related to the risk of developing postoperative complications, GLIM-defined malnutrition may better capture the patient’s ability to withstand and recover from surgical stress, thus influencing complication severity.

Rather than representing a contradiction, these findings underscore the complexity of morphofunctional assessment and highlight the importance of multivariable modelling in accurately capturing surgical risk.

The results of the current study have relevant clinical implications. First, they support the routine incorporation of body composition and nutritional assessment into preoperative evaluation in liver surgery. CT-derived SMI can be obtained from standard imaging without additional cost or burden, while GLIM criteria offer a structured and clinically applicable framework for diagnosing malnutrition. Second, both sarcopenia and malnutrition represent potentially modifiable risk factors. In this context, prehabilitation strategies combining exercise training, nutritional support, and metabolic optimization have shown promising results in improving postoperative outcomes. Notably, randomized evidence in liver surgery, including early trials [24] and more recent studies such as the PREHEP trial [12], has demonstrated that multimodal prehabilitation can reduce postoperative morbidity in high-risk patients undergoing liver resection. From a practical perspective, the integration of SMI and GLIM assessment into routine clinical workflows may enhance perioperative risk stratification and facilitate the identification of high-risk patients who could benefit from targeted interventions, including nutritional optimization and structured prehabilitation programs. This approach supports a more individualized perioperative management and aligns with the principles of enhanced recovery after surgery (ERAS) programs, which emphasize patient optimization and tailored perioperative care.

However, some limitations should be acknowledged. First, the retrospective design may introduce selection bias and limit causal inference. Second, some components of the GLIM criteria, such as unintentional weight loss, could not be systematically assessed due to incomplete data, potentially leading to an underestimation of malnutrition prevalence. Importantly, despite the absence of weight loss data, GLIM-defined malnutrition remained independently associated with severe postoperative morbidity. However, this limitation may have led to misclassification and underdiagnosis of malnutrition, potentially attenuating its association with postoperative outcomes. Third, functional and strength assessments, such as handgrip strength or physical performance tests, were not available, limiting the evaluation of sarcopenia as a multidimensional construct. Fourth, the cohort included a heterogeneous population with different underlying pathologies and indications for liver resection, which may influence both nutritional status and postoperative outcomes. However, this heterogeneity also reflects real-world clinical practice in a tertiary referral center, enhancing the external applicability of our findings. Finally, the use of cohort-specific SMI thresholds may limit the external generalizability of our findings and warrants validation in independent populations. In addition, the use of ROC-derived thresholds within the same dataset may introduce a degree of circularity and should be considered exploratory. Subgroup analyses were not performed due to limited statistical power.

## 5. Conclusions

Postoperative outcomes after liver resection are determined by a combination of perioperative factors, including patient-related morphofunctional vulnerability, surgical complexity, and postoperative inflammatory response. Reduced skeletal muscle mass independently predicts the occurrence of postoperative complications, whereas GLIM-defined malnutrition appears to be more closely associated with their severity.

These findings highlight the complementary role of body composition and nutritional assessment within a broader perioperative risk framework and support their integration into routine clinical practice. In addition, the identification of clinically relevant threshold values for perioperative variables may help improve risk stratification and early identification of high-risk patients.

Importantly, the identification of these potentially modifiable risk factors provides an opportunity to implement targeted prehabilitation strategies aimed at improving surgical resilience and postoperative outcomes.

Together, these results support a more comprehensive and individualized approach to perioperative risk assessment, integrating morphofunctional parameters alongside surgical and inflammatory factors.

## Figures and Tables

**Table 1:** Baseline clinical, nutritional and body composition characteristics.

	Total Patients (n = 253)	No Complications (n = 163)	Complications (n = 90)	*p*-Value
Clinical characteristics				
Age (years)	65 (58–73)	64 (25–84)	68.5 (42–85)	0.11
Male sex, n (%)	156 (61.7%)	102 (62.6%)	54 (60%)	0.69
CCI	7 (6–8)	7 (0–15)	6 (2–15)	0.81
ASA ≥ III, n (%)	150 (59.3%)	91 (55.8%)	59 (65.6%)	0.13
Nutritional status and body composition				
BMI, kg/m^2^	26.3 (23.6–29.7)	26.6 (15.2–42.9)	26 (19.1–38.3)	0.26
SMI, cm^2^/m^2^	45.3 (37.6–52.3)	43.7 (22.7–73.9)	46.6 (18.4–76.2)	0.08
SMM, cm^2^	124.5(100.9–149.5)	119.9 (54.4–218.7)	126.9 (47.9–187.9)	0.16
Low SMI (BMI ≥ 25), n (%)	142 (56.1%)	98 (60.1%)	44 (48.9%)	0.08
Low SMI (BMI ≥ 30), n (%)	126 (49.8%)	88 (54%)	38 (42.2%)	0.07
Sarcopenic obesity (BMI ≥ 25), n (%)	20 (7.9%)	15 (9.2%)	5 (5.6%)	0.30
Sarcopenic obesity (BMI ≥ 30), n (%)	30 (11.9%)	22 (13.5%)	8 (8.9%)	0.29
GLIM-defined malnutrition				
GLIM (BMI only)				
No malnutrition	223 (88.1%)	139 (85.3%)	84 (93.3%)	0.06
Moderate/severe malnutrition	30 (11.9%)	24 (14.7%)	6 (6.7%)	
Malnutrition (BMI + SMI, BMI ≥ 25), n (%)	149 (58.9%)	103 (63.2%)	46 (51.1%)	0.06
Malnutrition (BMI + SMI, BMI ≥ 30), n (%)	135 (53.4%)	94 (57.7%)	41 (45.6%)	0.06

CCI: Charlson comorbidity index; ASA: American Society of Anesthesiologists; BMI: body mass index; SMI: skeletal muscle index; SMM: skeletal muscle mass; GLIM: Global Leadership Initiative on Malnutrition. Statistics presented as median (range) or n (%).

**Table 2:** Tumour and surgical characteristics.

	Total Patients (n = 253)	No Complications (n = 163)	Complications (n = 90)	*p*-Value
Tumour characteristics				
Malignant disease, n (%)	236 (93.3%)	151 (92.6%)	85 (94.4%)	0.58
Colorectal liver metastases, n (%)	185 (73.1%)	120 (73.6%)	65 (72.2%)	0.83
Hepatocellular carcinoma, n (%)	49 (19.4%)	30 (18.4%)	19 (21.1%)	
Other diagnoses, n (%)	19 (7.5%)	13 (8%)	6 (6.7%)	
Number of lesions	1 (1–12)	1 (1–12)	1 (1–9)	0.48
Tumour size, mm	23 (4–180)	25 (4–150)	29 (10–150)	0.55
Posterior segments involvement, n (%)	93 (36.8%)	69 (42.3%)	50 (55.6%)	0.04
Lesions near major vessels, n (%)	115 (45.5%)	48 (29.4%)	33 (36.7%)	0.24
Preoperative embolization, n (%)	15 (5.9%)	4 (2.5%)	11 (12.2%)	0.004
Surgical characteristics				
Approach, n (%)				
Open	171 (67.6%)	97 (59.5%)	74 (82.2%)	<0.001
Laparoscopic	82 (32.4%)	66 (40.5%)	16 (17.8%)	
Major hepatectomy, n (%)	55 (21.7%)	30 (18.4%)	25 (27.8%)	0.08
Extrahepatic procedures, n (%)	62 (24.5%)	33 (20.2%)	31 (34.4%)	0.01
Operative time, minutes	210 (45–510)	210 (80–510)	240 (120–450)	0.001

Statistics presented as median (range) or n (%).

**Table 3:** Postoperative biomarkers.

	Total Patients (n = 253)	No Complications (n = 163)	Complications (n = 90)	*p*-Value
CRP (mg/L)				
POD1	128.9 (0.1–357.5)	173.85 (0.10–336.20)	101.35 (37.60–357.50)	0.37
POD3	121.4 (0.7–405)	198.30 (23.70–357)	135.75 (46.90–399.50)	0.02
POD5	87.4 (18.6–362.4)	94.65 (29.80–283)	92.75 (36.40–229.80)	0.06
Leukocyte count (×10^9^/L)				
POD1	10.5 (0.9–24.9)	10.8 (7.9–16.5)	11.5 (6.9–17.6)	0.61
POD3	8.6 (0.9–19.9)	9.2 (1.0–11.8)	9.1 (5.8–19.9)	0.67
POD5	7.7 (2.8–23.4)	8.7 (5.8–15.9)	7 (2.8–17.6)	0.6
Lymphocyte count (×10^9^/L)				
POD1	0.9 (0.2–2.8)	0.9 (0.7–1.5)	0.9 (0.4–1.8)	0.16
POD3	1.1 (0.06–1.55)	1.2 (0.1–2.1)	0.9 (0.5–1.5)	0.001
POD5	1.1 (0.2–3.4)	1.3 (0.3–2.3)	1 (0.7–2.6)	0.13
NLR				
POD1	9.4 (1.7–48.7)	8.4 (5.8–14.3)	10.9 (4.3–35.6)	0.03
POD3	6.1 (0.04–97.6)	5.4 (3.2–15.2)	6.7 (3.8–21.7)	0.02
POD5	4.6 (0.3–45.5)	5.2 (2.2–45.5)	4.2 (0.9–22.9)	0.79
PLR				
POD1	171 (48.7–1090)	152.2 (73–192.6)	155.8 (104.2–233.3)	0.93
POD3	148.23 (1.34–1600)	137.6 (48.9–1183.3)	150.9 (89.9–164.2)	0.05
POD5	173.3 (52–786.7)	173.9 (84–265.7)	160.6 (75.7–215.1)	0.39
LMR				
POD1	1.2 (0.3–7.1)	1 (0.5–2.4)	1.3 (0.3–2.3)	0.15
POD3	1.5 (0.02–287)	1.5 (0.9–3.3)	1.65 (0.9–2)	0.12
POD5	1.4 (0.02–6.6)	1.1 (0.6–2.8)	1.47 (0.7–3.5)	0.45

CRP: C-reactive protein; NLR: neutrophil-to-lymphocyte ratio; PLR: platelet-to-lymphocyte ratio; LMR: lymphocyte-to-monocyte ratio; POD: postoperative day. Statistics presented as median (range) or n (%).

**Table 4:** Postoperative outcomes.

Overall complications (90 days), n (%)	90 (35.6%)
Major complications (Clavien–Dindo ≥ III), n (%)	37 (14.6%)
Severe morbidity (CCI ≥ 26.2), n (%)	44 (17.4%)
Reintervention, n (%)	28 (11.1%)
Length of hospital stay, days	5 (1–90)
30-day mortality, n (%)	4 (1.6%)
90-day mortality, n (%)	4 (1.6%)

Statistics presented as median (range) or n (%).

**Table 5:** Multivariable analysis of postoperative complications at 90 days.

Variable	OR (95% IC)	*p*-Value
Low skeletal muscle index	2.23 (1.14–4.38)	0.02
Open surgical approach	3.14 (1.47–6.70)	0.003
Operative time ≥ 193 min	3.07 (1.58–5.98)	0.001
CRP ≥ 124 mg/L (POD3)	2.07 (1.10–3.91)	0.02

CRP: C-reactive protein.

**Table 6:** Multivariable analysis of major complications (Clavien–Dindo ≥ III).

Variable	OR (95% IC)	*p*-Value
Diabetes mellitus	2.80 (1.11–7.04)	0.02
Low skeletal muscle index	4.28 (2.76–10.40)	0.001
Tumour size ≥ 20.5 mm	3.20 (1.24–8.23)	0.01
Open surgical approach	4.89 (1.51–15.85)	0.008
Posterior segments involvement	2.78 (1.18–6.52)	0.01
Operative time ≥ 185 min	2.81 (1.05–7.47)	0.03
Lymphocytes POD3 < 0.7 ×10^9^/L	4.93 (1.90–12.78)	0.001

POD3: postoperative day 3.

**Table 7:** Multivariable analysis of severe morbidity (CCI ≥ 26.2).

Variable	OR (95% IC)	*p*-Value
GLIM-defined malnutrition	2.95 (1.44–6.06)	0.003
Posterior segments involvement	2.33 (1.13–4.80)	0.02
Major hepatectomy	2.46 (1.13–5.34)	0.02

GLIM: Global Leadership Initiative on Malnutrition.

## Data Availability

The data presented in this study are available on request from the corresponding author due to privacy and ethical restrictions.

## Supplementary Materials

The following supporting information can be downloaded at: https://www.mdpi.com/article/10.3390/jcm15103581/s1, Figure S1: ROC curve for the multivariable model predicting overall complications; Figure S2: ROC curve for major complications (Clavien–Dindo ≥ III); Figure S3: ROC curve for severe morbidity (CCI ≥ 26.2).
