# Supplementary material for: Perioperative Determinants of Postoperative Morbidity After Liver Resection: From Morphofunctional Vulnerability to Inflammatory Response
Source: J Clin Med. 2026 May 7;15(10):3581. doi: 10.3390/jcm15103581 (PMC13207638; doi:10.3390/jcm15103581)
Supplement: Supplementary file 1 [file jcm-15-03581-s001.zip › jcm-4291685-supplementary.pdf]

Supplementary Materials:

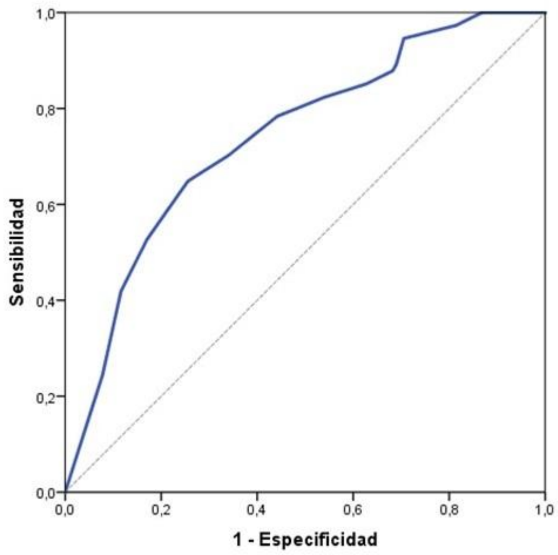

AUC (IC95%): 0,74 (0,67–0,81)
P< 0,001

Figure S1. ROC curve for the multivariable model predicting overall postoperative complications.

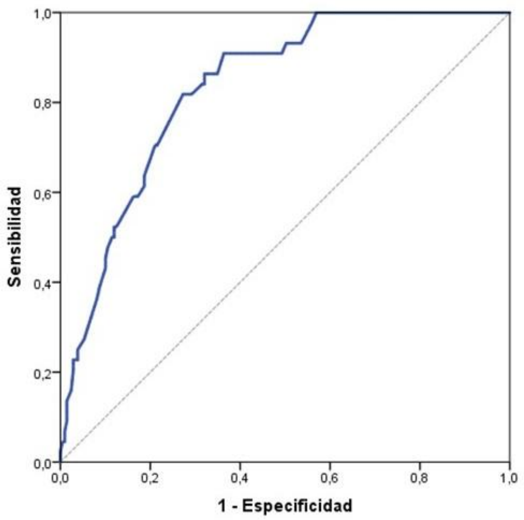

AUC (IC95%): 0,83 (0,78–0,89)
P< 0,001

Figure S2. ROC curve for the multivariable model predicting major complications (Clavien–Dindo ≥III).

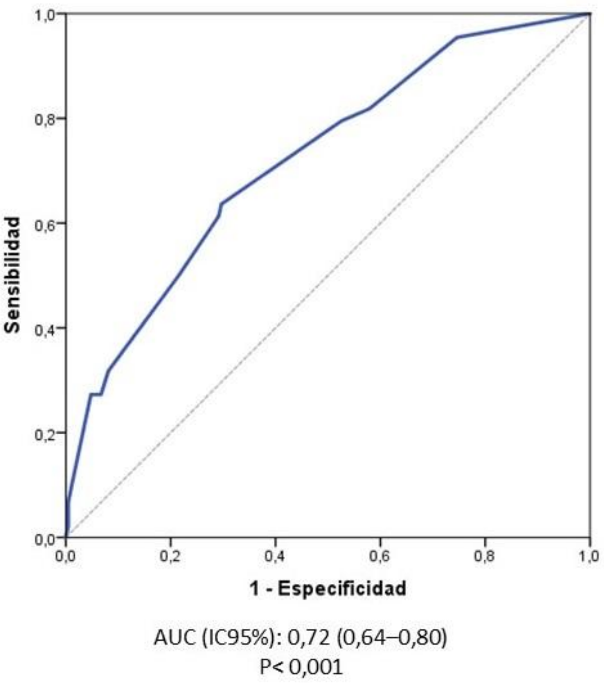

Figure S3. ROC curve for the multivariable model predicting severe morbidity (CCI ≥26.2).
